# Effect of [*n*]-Helicene Length on Crystal Packing

**DOI:** 10.1021/acs.cgd.3c00964

**Published:** 2023-10-24

**Authors:** Julia A. Schmidt, Emma H. Wolpert, Grace M. Sparrow, Erin R. Johnson, Kim E. Jelfs

**Affiliations:** † Department of Chemistry, Imperial College London, Molecular Sciences Research Hub, White City Campus, Wood Lane, London W12 0BZ, U.K.; ‡ Department of Chemistry, Dalhousie University, Halifax, Nova Scotia B3H 4R2, Canada

## Abstract

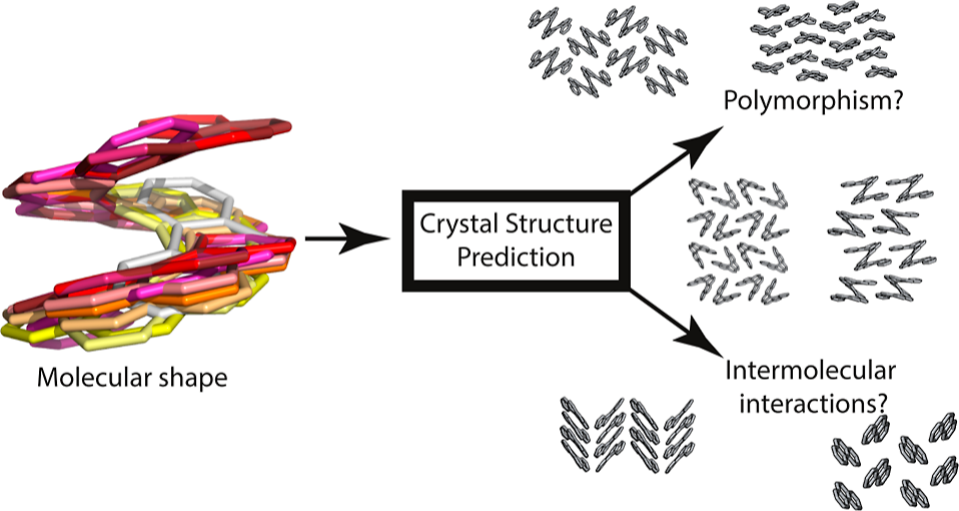

Chiral π-conjugated organic molecules hold potential for emerging technologies as they are capable of introducing novel functionalities into electronic devices owing to their strong chiroptical properties. However, capitalizing on chiral molecules for electronic devices is reliant on their molecular packing—a factor that impacts their charge-transport properties. The solid-state behavior of molecules is sensitive to subtle differences in molecular interactions, chirality, and shape, but these relationships are not fully understood. Here, we employ crystal structure prediction (CSP) as a tool to probe the lattice-energy landscape for a family of chiral organic molecules: [*n*]helicenes, where *n* ranges from 3 to 12. Our results show excellent agreement between the CSP landscapes and experimentally reported structures. By analyzing the packing motifs within the polymorph landscapes, we begin to develop an understanding of how helicene length affects the shape and π–π stacking interactions seen in the polymorphs. Furthermore, we propose how helicene length can be used as a tool to design new functional organic electronics.

## Abstract 2

Chiral π-conjugated organic molecules hold potential for emerging technologies as they are capable of introducing novel functionalities into electronic devices owing to their strong chiroptical properties.

## Introduction

Organic semiconductors (OSCs) are low-cost, environmentally friendly alternatives to inorganic semiconductors. With their potential to usher in a new era of sustainable technologies, they are being explored extensively in a diverse variety of applications.^1−3^ Moreover, due to their flexibility and biodegradability, they provide new functionalities to semiconducting materials such as flexible computer screens,^4−6^ biodegradable electronics (e.g., disposable phones),^4,7,8^ and energy-harvesting smart materials. The device performance strongly depends on the ease with which charge carriers move from one molecule to another, which is determined by the solid-state arrangement of the molecules.^9^ OSCs tend to be polymorphic, existing in multiple crystalline packings under ambient conditions. Different crystalline structures, sometimes only with marginal changes to the crystal packing, can have considerably different properties,^1,9−11^ and consequently polymorphism can be used as a design strategy for high-performance organic electronics.^9^

Introducing chirality into OSCs adds another level of functionality as it enables the production of polarization-selective photodetectors,^12^ chiroptical switches,^13−15^ and room-temperature spintronic devices.^16^ Carbo[*n*]helicenes (referred to throughout this work as [*n*]helicenes) form a family of archetypal chiral π-conjugated organic molecules that are formed from ortho-fused, angularly arranged benzene rings, where *n* is the number of aromatic rings in the molecule. Helicenes are promising compounds for designing new organic electronic technologies due to their large chiroptical response, charge-transport properties, and their ability to filter electron spin at room temperature.^17−20^ Chirality adds another level of complexity to solid-state formation, as chiral compounds can form both enantiopure and racemic crystals with very different electron transport properties. For example, aza[6]helicene has an 80-fold increase in hole mobility in the racemate structure compared to the enantiopure structure.^19^

Despite the interest in helicenes, their application in electronic devices remains in its infancy, partly due to a poor understanding of molecular packing and how it impacts performance. Much of our own work has looked to bridge this gap using crystal structure prediction (CSP) as a means to develop our understanding of structural motifs that enhance optoelectronic properties. CSP is particularly useful for these molecules due to the inherent rigidity of the structures. In fact, both naphthalene and benzene are used for testing and benchmarking CSP protocols.^21,22^ CSP of helicenes in our own work has helped predict the thin-film packings of enantiopure and racemic aza[6]helicene, explaining 80-fold differences in charge mobility.^19^ Combining CSP with the prediction of optoelectronic properties can be used to develop energy–structure–function (ESF) maps.^23−25^ We have used this framework to explore the ESF of [6]helicene using CSP.^26^ This work highlighted specific structural motifs that were particularly beneficial for either high electron or hole mobility. In an effort to enhance our understanding of the interplay between molecular structure and optoelectronic properties, we have investigated the impact of altering the nitrogen atom’s position within aza[6]helicene, while maintaining identical crystal packing.^27^ Our findings show that the position of nitrogen can be crucial in determining the semiconducting properties of the material. Using this prior knowledge of how changes in the functional group position and molecular assembly separately affect the charge-carrier mobilities, we screened over 1300 substituted [6]helicenes for high charge mobility.^28^ This approach shows promise in guiding the design of new OSC molecular materials and identified fluorinated [6]helicenes as being the most promising to maximize the OSC performance metrics. However, it is difficult to outperform unsubstituted helicenes in terms of their high charge mobility.

**Figure 1:**
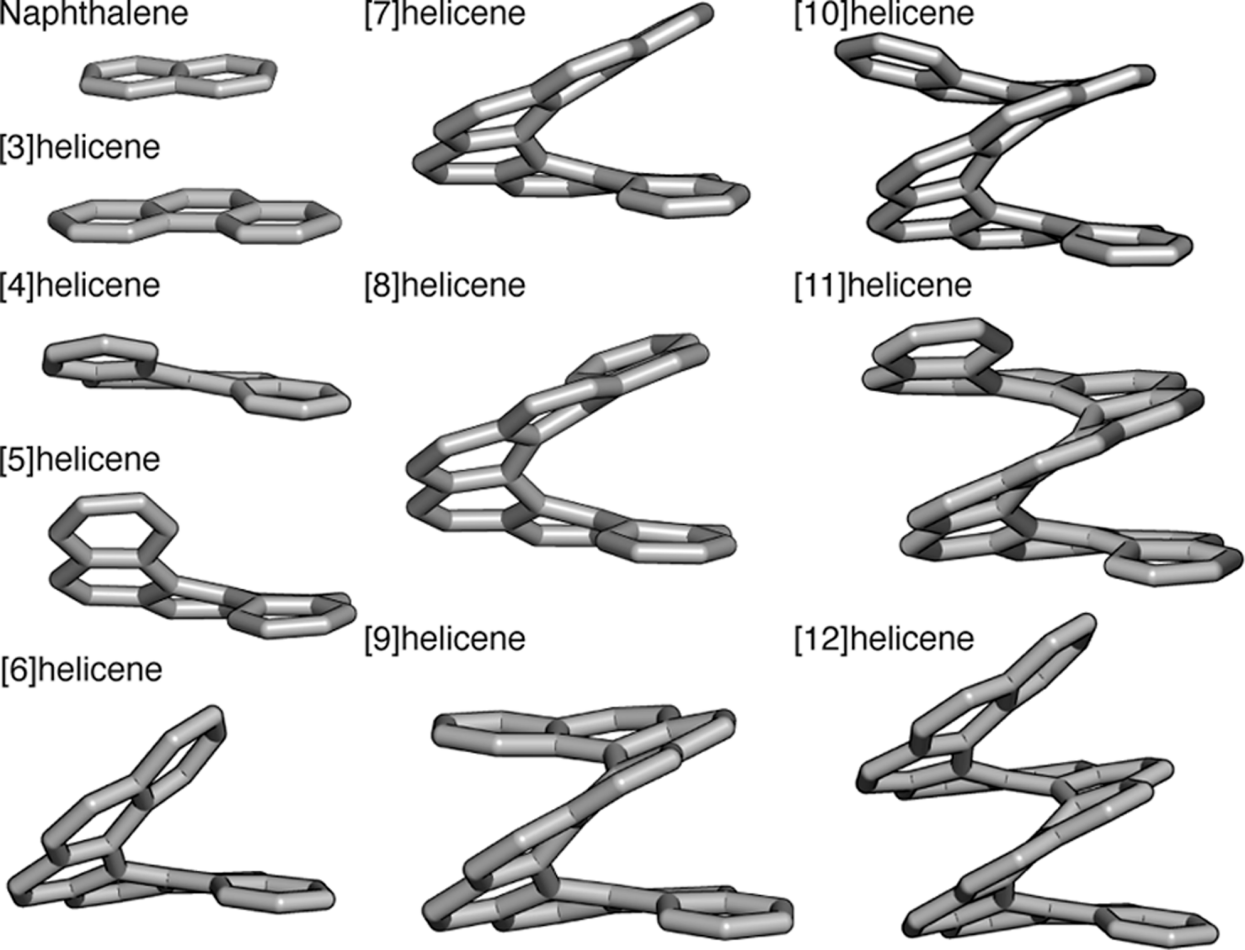
Molecular structures of naphthalene and [*n*]helicenes of varying helicene length *n*, here *n* = 3–12. Hydrogens are omitted for clarity.

Each of these studies has provided insights into how the supramolecular packing of helicenes affects their properties. Here, we aim to build on these studies by exploring the relationship between molecular shape, chirality, size, and intermolecular interactions and their effect on crystal packing. Through this study, we use CSP to explore the effect of changing the number of aromatic rings, *n*, in [*n*]helicene on the molecules’ crystal packing behavior and intermolecular interactions. Using the CSP landscapes of naphthalene and [3]–[12]helicene (Figure 1), we examine the trends in the polymorphic behavior and π–π interactions. Our results show that the packing behavior changes as a function of the molecular shape, with similar length helicenes containing similar packing motifs. Moreover, the lattice energy increases with the degree of π–π stacking in the crystal, but mid-length helicenes ([5]–[7]helicene) are more likely to contain stable structures with some degree of π–π stacking than other helicenes. For [*n*]helicenes whose crystal structures have been obtained experimentally, our results show excellent agreement between the computationally predicted lowest energy structure and that obtained experimentally. By analyzing the computationally predicted structures for [*n*]helicenes whose crystal structures have not been characterized, we posit that [12]helicene may have high hole mobility, making it an attractive candidate for organic electronic devices.

## Methods

### Crystal Structure Prediction

The naphthalene and [*n*]helicene molecules (*n* = 3–12) were geometry optimized using Gaussian 16^29^ at the B3LYP^30^/6-31G(d,p) level of theory, with tight convergence criteria and assuming no symmetry. Based on the optimized geometry, the charges were computed from the electrostatic potentials using a grid-based method (ChelpG).^31^ The CrystalPredictor II^32,33^ software package was used to generate hypothetical crystal structures within a polymorphic region spanning an energy range of up to 20 kJ mol^–1^ above the global minimum using pairwise potential parameters. Here, the molecules were treated as rigid, and the search was restricted to only one molecule in the asymmetric unit (*Z*′ = 1) to limit the computational expense. Although limiting the search space to *Z*′ = 1 may lead to an under prediction of experimentally realized structures, extending the search to *Z*′ = 2 is likely to just produce closely related and duplicated structures of those found in the *Z*′ = 1 landscape. Moreover, the *Z*′ = 1 CSP search can provide many insights about the possible crystal packings of the molecule, which was our primary aim of this study.^34^ Next, 500,000 crystal minimizations were performed across the following space groups: *P*1, *P*1̅, *P*2_1_, *P*2_1_/*c*, *P*2_1_2_1_2, *P*2_1_2_1_2_1_, *Pna*2_1_, *Pca*2_1_, *Pbca*, *Pbcn*, *C*_2_/*c*, *Cc*, *C*2, *Pc*, *Cm*, *P*2_1_/*m*, *C*2/*m*, *P*2/*c*, *C*222_1_, *Pmn*2_1_, *Cmc*2_1_, *Aba*2, *Fdd*2, *Iba*2, *Pnna*, *Pccn*, *Pbcm*, *Pnnm*, *Pmmn*, *Pnma*, *Cmcm*, *Cmca*, *Fddd*, *Ibam*, *P*4_1_, *P*4_3_, I4̅, *P*4/*n*, *P*4_2_/*n*, *I*4/*m*, *I*4_1_/*a*, *P*4_1_2_1_2, *P*4_3_2_1_2, *P*4̅2_1_*c*, I4̅2d, *P*3_1_, *P*3_2_, *R*3, *P*3̅, *R*3̅, *P*3_1_21, *P*3_2_21, *R*3̅*c*, *R*3*c*, *P*6_1_, *P*6_3_, *P*6_3_/*m*, *P*2_1_3, *Pa*3̅, *P*222_1_, and *Pba*2. All space groups were then searched in proportion according to their relative abundance in the CSD,^32^ and all the stable crystal structures were clustered to remove duplicates using the CCDC COMPACK^35^ module.

The tentative crystal structures obtained from CrystalPredictor were relaxed using distributed multipoles and the W99 parameters for the repulsion-dispersion potential within DMACRYS.^36,37^ A subset of the force-field optimized structures that were within 10 kJ mol^–1^ per molecule of the minimum were then fully relaxed and reranked using density-functional theory (DFT) with the FHI-aims program.^38^ The DFT calculations used the B86bPBE density functional^39,40^ and the exchange-hole dipole moment (XDM) dispersion model,^41,42^ with “light” basis sets and “tight” integration grids. The DFT-optimized structures can be found at https://github.com/ewolpert1/nhelicenes. The 10 kJ mol^–1^ energy cut off was selected from considerations of the previous six helicene structures studied,^19,26,28^ plus compounds from the first five CSP blind tests.^43−47^ The threshold selected corresponds to the 95% confidence interval that the minimum-energy structure will be included and carried forward to the DFT relaxations. Furthermore, structures were identified as duplicates and removed if they were within 0.01 kJ mol^–1^ in energy and 0.01 g cm^–3^ in unit-cell density. These values were selected as conservative cutoffs since duplicate structures can possess energy and volume differences larger than this due to the choice of geometry relaxation thresholds. We note that B3LYP slightly over stabilizes extended conjugation, and using it as the functional for the initial geometry optimization of the helicene molecule may result in small deviations from the helicene’s ideal (gas phase) geometry. However, this is not expected to significantly impact the structures generated through DMACRYS, or their relative energies, such that the lowest energy polymorphs lie within the 10 kJ mol^–1^ cutoff used for structures that then undergo reoptimization with FHI-aims.

Additional DFT geometry optimizations were performed on experimental crystal structures of the [*n*]helicene compounds taken from the Cambridge Structural Database (CSD) in cases where a matching crystal structure was not generated by the quasi-random search. These were enantiopure structures of [7]helicene and [9]helicene (IMEJIW with *Z*′ = 2 and QUJNEQ with *Z*′ = 3) and racemic structures of [5]helicene (DBPHEN03 with *Z*′ = 2 and DBPHEN04 with *Z*′ = 3). For the intergrowth structure of [6]helicene, the crystal structure was taken from ref (26).

### Crystal Packing Analysis

The crystal packing analysis was performed using CRYSTACK, an open-sourced python module https://github.com/juliaaschmidt/crystack, developed here for the analysis of π–π stacking interactions. The analysis is performed by building a 4 × 4 × 4 supercell, taking the central molecule and calculating its intermolecular interactions with molecules within the first neighboring shell. Around the most central molecule, the center-of-mass to center-of-mass distance to all surrounding molecules is computed. As CRYSTACK only considers the central molecule, this code works for *Z*′ = 1 polymorphs only. The five unique dimers with the shortest center-of-mass to center-of-mass distance are extracted from the shell (ignoring duplicate dimers). For each dimer, the CRYSTACK module computes all molecule–molecule interactions.

The focus is on π–π interactions due to their relevance to organic semiconducting devices. For each crystal in the 11 CSP landscapes, we calculated the extent of parallel π–π stacking interactions for each molecule expressed as a percentage per molecule. Aromatic rings within the molecule were only considered to be π-stacked if the intermolecular distance between the center of the benzene rings did not exceed 5.5 Å, the angle between the benzene rings was within 0–30°, and the displacement between the centroids of the two benzene rings parallel to the plane of the rings was less than 2.0 Å.

## Results and Discussion

### Comparison to the Experiment

**Figure 2:**
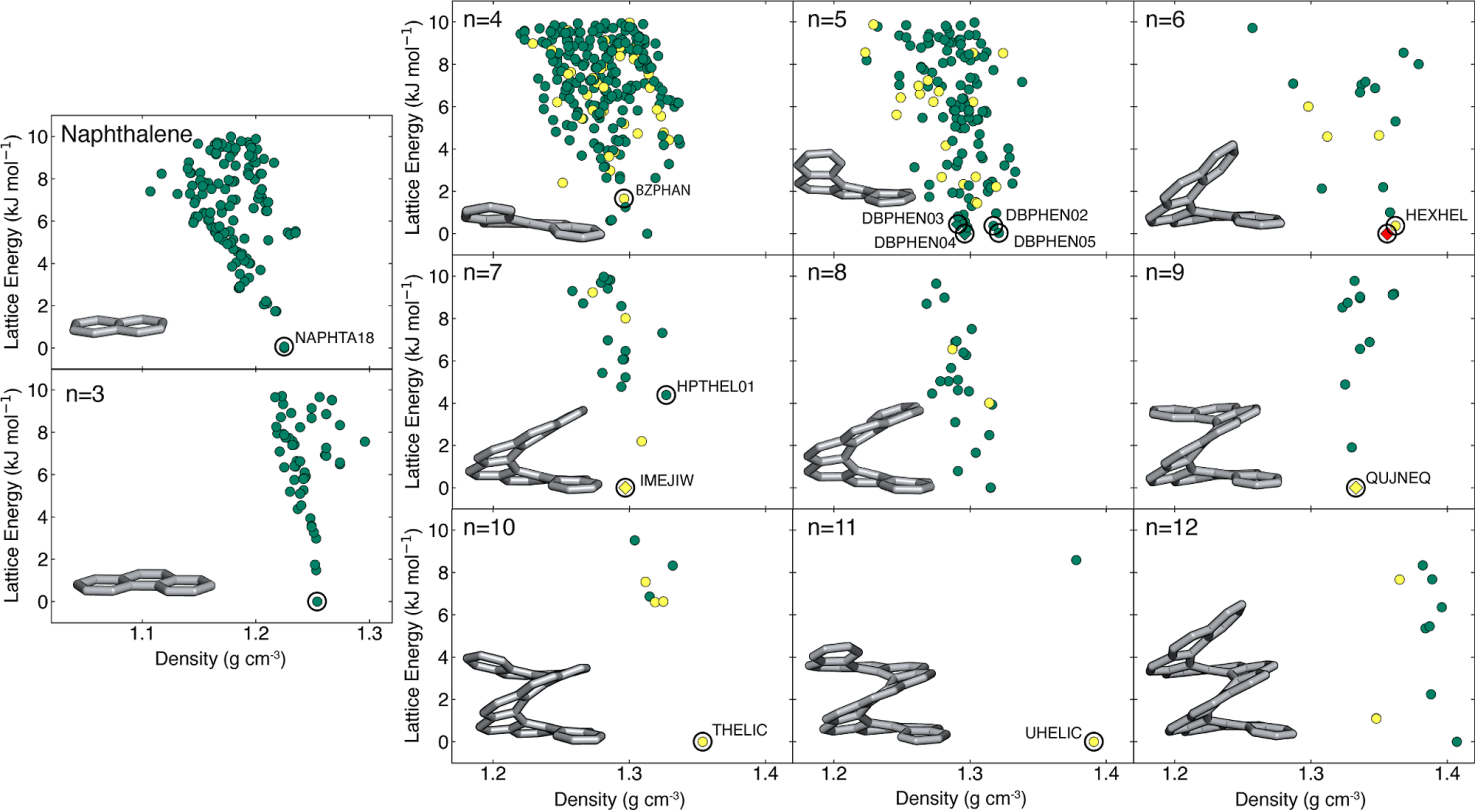
CSP landscapes for naphthalene and [3]–[12]helicenes for the polymorphs within 10 kJ mol^–1^ of the global minimum. Each data point is colored by the chirality of the crystal structure, either in yellow (enantiopure) or teal (racemic). The intergrowth crystal structure of [6]helicene is shown in red. Black circles indicate the experimentally observed polymorphs. If the experimental structure had *Z*′ > 1, the structure is represented as a diamond rather than a circle. CSD codes are provided next to the corresponding structures when available.

**Figure 3:**
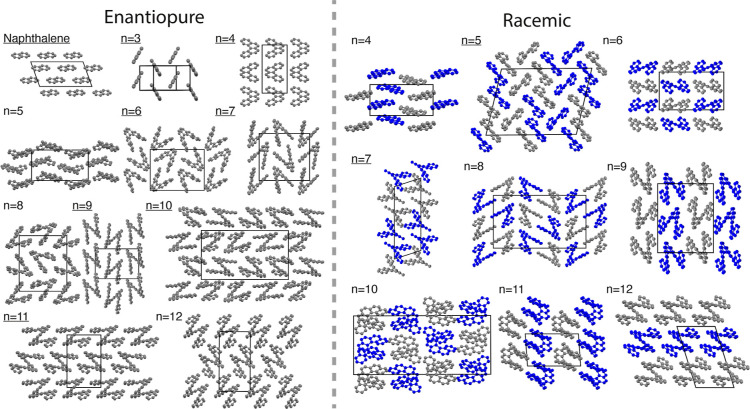
Lowest energy enantiopure and racemic polymorphs for naphthalene and [3]–[12]helicenes as found in the CSP landscapes. Polymorphs that have been experimentally synthesized are underlined. For [3]helicene, there is no reported structure in the CSD, but the experimental structure was obtained by using the crystal structure data provided in ref (48) to generate a CIF file, which was then overlaid with predicted structures of similar space group and crystal parameters. For the enantiopure structures from *n* = 2, 4, 6, 7, 9, 10, and 11, the synthesized polymorphs correspond to crystal structures with the CSD codes: NAPHTA18, BZPHAN, HEXHEL, IMEJIW, QUJNEQ, THELIC, and UHELIC. For the racemic structures from *n* = 5–7, the synthesized polymorphs correspond to crystal structures with the CSD codes: DBPHEN04 and HPTHEL. The gray and blue helicenes in the racemic crystals indicate the two enantiomers.

Of the molecules studied in this paper, crystal structures of naphthalene and [*n*]helicenes with *n* = 3–7 and 9–11 have been synthesized^48−60^ with more than one polymorph obtained for [5]helicene^49−52^ and [7]helicene.^55,56^ With the exception of [3]helicene, X-ray crystal structures were deposited to the CSD, which we used to compare with the structures within our CSP landscapes. We found that the CSP landscapes (Figure 2) were able to reproduce most of the experimentally known crystal structures. In instances where this was not the case (*n* = 5, 6, 7, and 9), the experimental structure was either an intergrowth (as with [6]helicene, where the crystal structure contains alternating layers of the opposite helicene enantiomer^61^) or possessed a higher number of molecules in the asymmetric unit. For example, [5]helicene has two polymorphs (DBPHEN03 and DBPHEN04) where *Z*′ = 2 and 3, respectively, [7]helicene has one polymorph (IMEJIW) where *Z*′ = 2, and [9]helicene has one polymorph where *Z*′ = 3 (QUJNEQ). By construction of our approach, polymorphs with *Z*′ > 1 could not be found here as CSP searches with are significantly more computationally expensive. Calculating the energies of these experimental structures and including them in our CSP landscapes lead to the correct assignment of the experimental structure as the lowest energy structure (Figure 2). Representative configurations of the low-energy polymorphs—both enantiopure and racemic—for each of the CSP landscapes are shown in Figure 3.

If a *Z*′ = 1 structure had been experimentally realized (with the exception of [4]- and [7]-helicene, which will be discussed in detail), our B86bPBE-XDM calculations correctly identified the structure as the lowest energy *Z*′ = 1 polymorph within the landscape (Section S1) (Figure 2). Due to the nature of the noncovalent interactions between the molecules, use of DFT is critical for the correct assignment of polymorphs as calculations using DMACRYS often ranked the experimental polymorph higher in energy (Section S2). The only case where an experimentally realized *Z*′ = 1 polymorph was not produced in the generation of crystal structures is for [5]helicene, where the racemic DBPHEN02 is missing. However, another synthesized *Z*′ = 1 racemic polymorph, DBPHEN05, is predicted and is lower in energy than DBPHEN02.

For [4]helicene, the landscape contains three polymorphs with *Z*′ = 1 that are lower in energy than the experimental *Z*′ = 1 polymorph. This is because the lower energy polymorphs are racemic, whereas the experimentally realized crystal structure is enantiopure. Although the activation energy barrier to enantiomer interconversion for [4]helicene is small at ≈17 kJ mol^–1^,^62^ the racemic structure is only 1.66 kJ mol^–1^ lower in energy than the enantiopure structure; thus, the racemic structure is unlikely to form from an enantiopure solution, which we presume was used to crystallize [4]helicene,^60^ resulting in the crystallization of the enantiopure polymorph. We would expect that if [4]helicene were to be synthesized from a racemic mixture, then the lowest energy polymorph predicted in the landscape would form. Although it has been reported that [4]helicene forms a conglomerate crystal,^63,64^ we were (a) not able to access the crystal structure and (b) check the synthesis route for solvent effects as ref (64) did not report them. Therefore, we assume that desolvated racemic mixtures would form a racemic crystal.

For [7]helicene, only one *Z*′ = 1 polymorph is lower in energy than the experimental *Z*′ = 1 structure, and this discrepancy can be explained by the chirality once again. The experimental structure is racemic, whereas the lowest energy *Z*′ = 1 predicted structure is enantiopure. Typically, compounds that have a lower-energy enantiopure structure tend not to form racemic crystals, instead forming twinned crystals of different chirality, as seen for [6]helicene.^26^ However, for [7]helicene, the lowest energy racemic structure falls within the polymorphic region (<7.2 kJ mol^–1^ above the global minimum in 95% of cases),^65^ and its formation may be favored due to kinetic reasons. The enantiopure *Z*′ = 1 structure has not been realized experimentally, likely due to the global minimum corresponding to the enantiopure experimental polymorph (*Z*′ = 2), which is thermodynamically favored.

As well as the crystal structures reported in the literature discussed above, conglomerate crystals have been reported to form for [4]–[9]helicene.^63,64,66−69^ The formation of conglomerates (where crystals of the two enantiomers are formed in equal amounts) indicates that the enantiopure polymorph is more energetically stable than racemic crystal structures that might otherwise form from a racemic mixture. Conglomerate formation for [6], [7], and [9]helicene is consistent with our results as it confirms that enantiopure polymorphs are more stable than racemic polymorphs, as seen in our landscape. However, it is inconsistent with our predictions for [4], [5], and [8]helicene, where our CSP landscape predicts a racemic polymorph to be lower in energy than enantiopure polymorphs. The observation of these conglomerate crystals does not necessarily contradict our results as other factors, such as solvation effects, may affect crystal formation. For instance, the conglomerate structures of [5] and [8]helicene were observed when crystallizing from a solution of ethanol, and benzene and iodine, respectively. Additionally, the formation of stable racemic crystals of [5]helicene further implies that factors such as kinetics or solvation might play a role in the formation of the conglomerate structure as racemic polymorphs should not be accessible if an enantiopure crystal structure is lower in energy. The paper referring to [4]helicene conglomerates does not reference the synthesis route and so we are unable to offer similar comparisons.^64^

### Global Trends

Given the correlation between low-energy structures in the CSP landscapes and their experimental counterparts, we analyzed the packing motifs in the lowest-energy polymorphs for both enantiopure and racemic structures for naphthalene and [3]–[12]helicene to look for similarities between the packing behavior of the polymorphs with different number of aromatic rings (Figure 3). For the enantiopure structures, [4]helicene exhibits both herringbone-type packing and translational chains of helicenes that are similar to the adjacent translational chains as established in ref (26), whereas [5]helicene does not exhibit any packing motifs seen in the CSP landscape of [6]helicene. The [6], [7], and [8]helicenes are dominated by chains of interlocked pairs, whereas for *n* > 8, this packing behavior changes, and there is less interlocking between neighboring helicenes.

The racemic structures show more variation in packing behavior, where interlocked chains are not formed in any of the low-energy polymorphs. Instead a herringbone-type packing is seen in structures where *n* = 3, 5 and adjacent translational chains are seen in naphthalene and [6]helicene. The polymorph of [4]helicene is very similar to the enantiopure [5]helicene. [7]helicene is the only molecule that contains motifs similar to the interlocked enantiopure helicenes as the helicenes pack in the same back-to-back manner, whereas [8], [9], and [10]helicene have similar packing behavior to each of their enantiopure counterparts.

**Figure 4:**
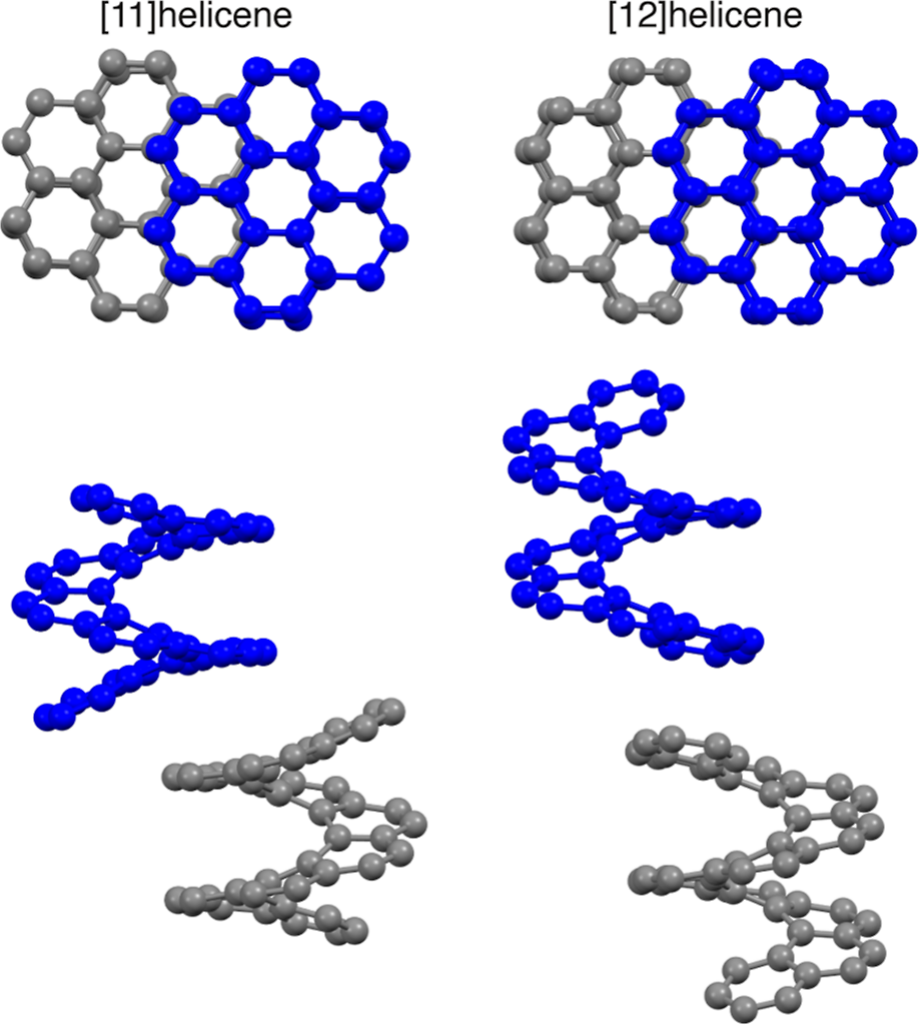
Packing motifs of lowest energy racemic structures of [11] and [12]helicene, which correspond to a motif that has high hole mobility in [6]helicene. The gray and blue colors of the helicenes indicate the two enantiomers.

The packing motifs change for [11] and [12]helicene where there are π-stacked chains of alternating enantiomers (Figure 4). These motifs are most likely seen in these helicenes rather than mid-length helicenes as there is a (near) full rotation of the helix, which promotes end-to-end terminal ring π–π interactions. Comparing these motifs to those found in [6]helicene shows that [11] and [12]helicene contain structural motifs that are similar to motifs that consistently resulted in calculated hole mobilities greater than 1 cm^2^ V^–1^ s^–1^ in [6]helicene.^26^ For [11]helicene, the racemic structure is substantially higher in energy than the global minimum enantiopure structure (above the polymorphic window) and so is unlikely to form. However, the racemic polymorph of [12]helicene is the global minimum, and so perhaps a solid form of [12]helicene may exhibit a similar high hole mobility as calculated for the [6]helicene polymorphs, making it a good candidate for charge mobility testing.

**Figure 5:**
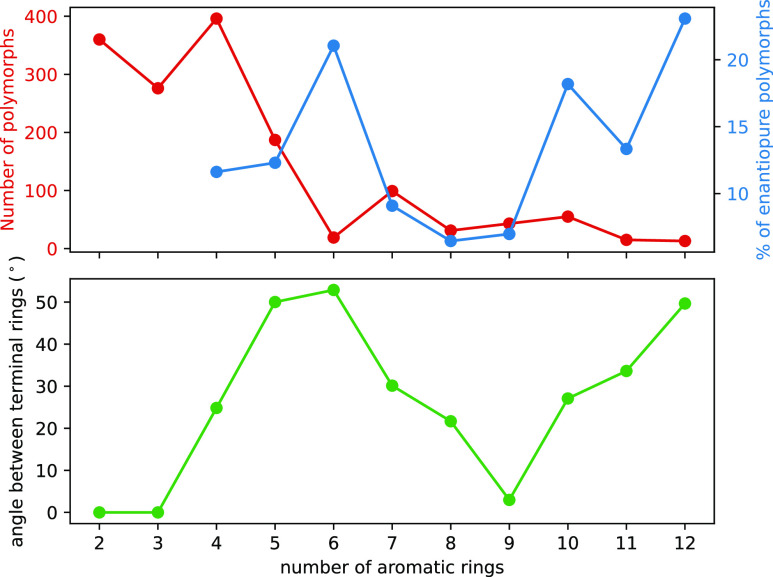
Number of polymorphs (red), the percentage of which are enantiopure (blue), and angle between terminal rings (green), for naphthalene and each helicene structure within 20 kJ mol^–1^ of the global minimum from each CSP search. As naphthalene and [3]helicene are not chiral, the % of enantiopure crystals has been omitted.

From the CSP search of naphthalene and [3]-[12]helicenes, the number of stable polymorphs obtained decreases with increasing helicene length (Figure 5). This phenomenon can be attributed to the fact that the longer the spiral shape of the molecule, the steeper the potential energy surface. This is likely due to the decreased directionality of interactions afforded by the smaller molecular shapes. For example, large molecular cages have relatively fewer low-energy polymorphs within their crystal energy landscape compared to smaller molecules such as benzene.^70,71^ Consequently, this reduces the configurational space of feasible solid-state formations.

The most significant decrease in the number of polymorphs occurs when increasing the number of aromatic rings from 4 to 6. We attribute this trend to the changing shape of the molecule from [4] to [6]helicene, wherein the molecule adopts a quasi-flat shape for *n* = 4, but becomes increasingly helical on increasing *n*, thereby disrupting some of the stable interactions and thus polymorphs available to (quasi-)flat aromatic compounds. The change in shape of the helicenes with changing number of aromatic rings can be quantified by the change in angle between the terminal aromatic rings, as shown in Figure 5. A similar but less pronounced decrease in the number of low-energy polymorphs is observed when *n* increases from 10 to 12, again likely due to the disruption of stable interactions when increasing the angle between the terminal rings. When the angle between the terminal rings is small, such as when *n* = 9, the molecule fills a more uniform space, leading to a larger number of similar packing motifs being energetically accessible. Conversely, the packing for helicenes with larger angles between terminal rings, such as [6] and [12]helicene, results in relatively fewer polymorphs. Interestingly, the proportion of enantiopure structures in the landscapes is highest for *n* = 6 and *n* = 12, implying that large angles between terminal rings, and thus nonuniform shapes, stabilize enantiopure structures relative to helicenes with more uniform shapes (Figure 5). This suggests that the stabilizing interactions that are lost when the molecular shape becomes less uniform occur predominantly in racemic structures rather than enantiopure.

**Figure 6:**
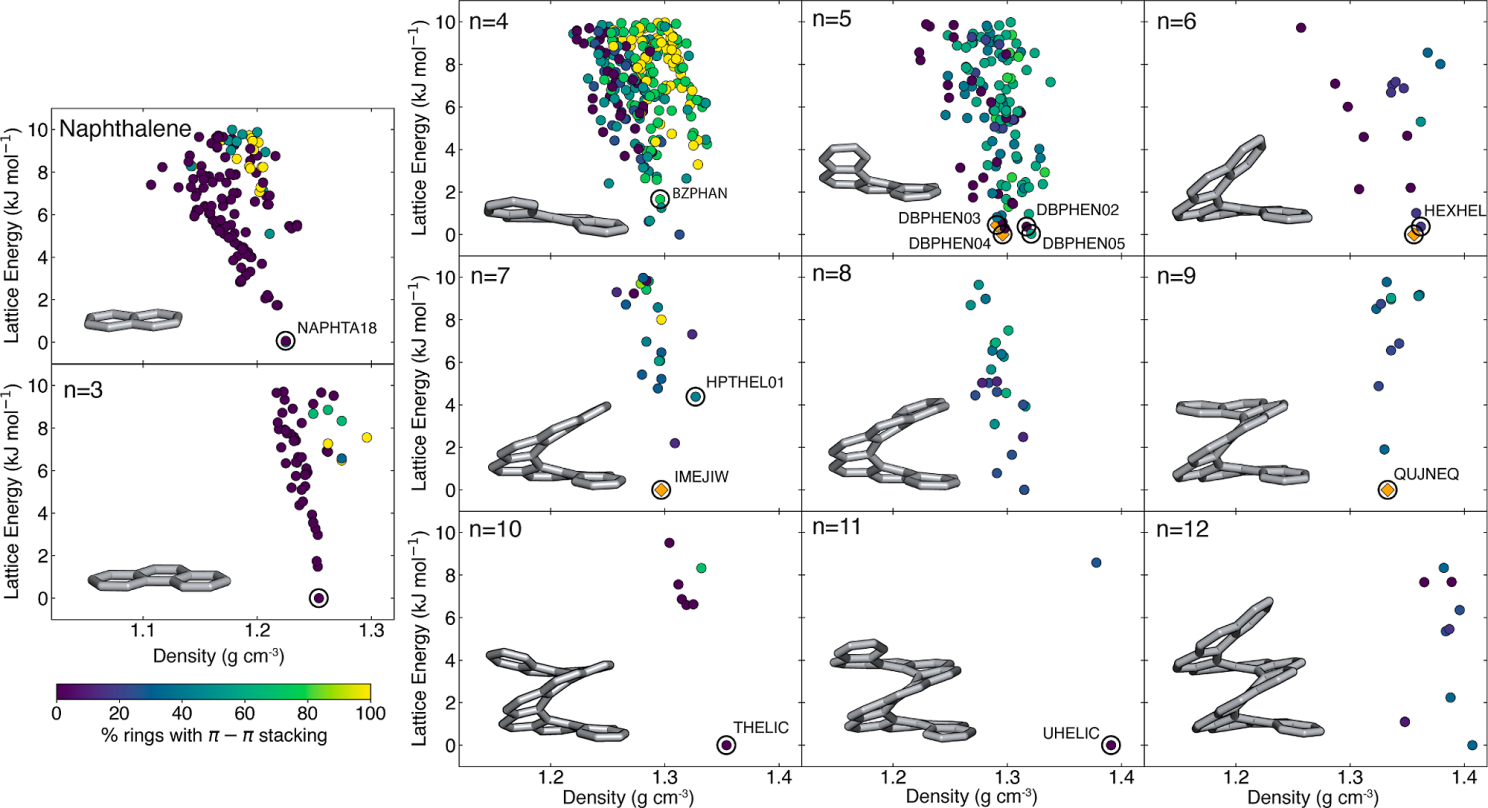
CSP landscapes for naphthalene and [3]–[12]helicenes for the polymorphs within 10 kJ mol^–1^ of the global minimum. Each data point is colored by the degree of π–π stacking of the single molecule in the asymmetric unit of the crystal. 100% (0%) denotes that every (no) benzene ring of the respective backbone is involved in π–π stacking. Black circles indicate experimentally observed polymorphs. If the experimental structure had *Z*′ > 1, the structure is represented as a diamond rather than a circle and colored orange as CRYSTACK is unable to calculate the extent of π–π stacking. CSD codes are provided next to the corresponding structure where available.

Given the agreement between the CSP landscapes and experimental structures, we proceeded to analyze the intermolecular interactions present within the polymorph landscapes. Part of the motivation behind studying the CSP landscapes of [*n*]helicenes is to gain an understanding of how changing the number of aromatic rings in the molecule impacts its packing behavior in the solid state. Through this analysis, we can build an understanding of how helicene length can be used in developing electronics with favorable properties. Carbohelicenes have two main noncovalent interactions between the molecules: C–H–π, and π–π interactions. As favorable charge-transport properties often rely on having a high degree of π–π stacking, our main interest lies in how the degree of π–π stacking changes with helicene length. In order to determine how helicene length might affect charge-transport properties, we evaluated the propensity for π–π stacking interactions for each helicene length by measuring the degree of π–π stacking in the polymorphs, quantified by the percentage of aromatic rings of the helicene backbone involved in a π–π interaction (Figure 6).

To identify any trends between π stacking interactions and lattice energy, we computed the Pearson correlation coefficients for each chain length (Figure S9). The results revealed a direct but weak relationship between π–π stacking interactions and crystal lattice energy for most values of *n*. Therefore, π–π stacking interactions are predominantly observed across higher-energy crystals and less frequently observed for low-energy crystals. However, on increasing chain length for naphthalene up to [6]helicene, π–π stacking becomes less unfavorable, to the point where there is no discernible correlation between the lattice energy and π–π stacking interactions, after which the correlation increases again, becoming maximally unfavorable for [8]helicene (Figure S9). The correlation between π–π interactions and the lattice energy then decreases as *n* increases, reaching a local minima for [11]helicene. These findings suggest that having a less uniform shape, as quantified by the large angles between terminal helicenes, may foster more favorable π–π stacking compared to helicenes with more uniform shapes. This is likely due to the direct relationship between the angle between the terminal rings and the helical rotation of the helicenes, as the larger angles for [6] and [12]helicene correspond to one and two full rotations of the helix, respectively. Therefore, for intermediate rotations of the helix, the nonterminal rings are not accessible for intermolecular π–π stacking.

It is crucial to note that a low Pearson correlation coefficient does not imply that π–π stacking is inherently favorable. In fact, the low Pearson correlation coefficient for [5]–[7]helicenes suggests that there is almost no correlation between π–π stacking and lattice energy and, thus, π–π interactions are neither favorable or unfavorable. Instead, in comparison to the higher Pearson correlation coefficients for other values of *n*, it suggests that π stacking is less unfavorable when the molecular shape is less uniform. This trend aligns with our initial observation of relatively fewer polymorphs in the CSP landscape for [6]helicene. The absence of a clear trend between π–π interactions and energy, i.e., the lack of these interactions predominately in higher energy crystals, suggests that other intermolecular interactions that typically stabilize the crystal structures for other [*n*]helicenes may be destabilized in [6]helicene. This observed trend may be the reason why chiral columns have been successfully synthesized experimentally in substituted [6]helicenes.^72,73^ Consequently, these trends could provide valuable insights into determining whether helicene is suitable for further polymorph studies or those whose derivatives may benefit from additional investigation.

## Conclusions

We used CSP to investigate the effect of changing helicene length on the polymorphism and intermolecular interactions for naphthalene and the [*n*]helicenes where *n* = 3–12. Features such as interlocked pairs with back-to-back packing are more often found in the low-energy enantiopure polymorphs rather than racemic polymorphs, whereas herringbone packing is more common in racemic structures. The polymorphic behavior changes significantly with changing *n*, with similar packing motifs found in helicenes of similar lengths. We suggest that the grouping of similar packing types is due to the similar shapes, which we quantify using the angle between the terminal aromatic rings. This measure also loosely correlates with other global trends seen in the CSP landscapes, such as the number of polymorphs and percentage of enantiopure structures. A key result from the analysis of the low-energy polymorphs is that the most stable polymorph of the uncharacterized [12]helicene crystal exhibits a packing motif that may have high hole mobility, indicating efficient charge transport and enhanced device performance.

Using computational studies to explore the polymorphic landscapes of organic electronics helps improve our understanding of the underlying factors driving the packing behavior and can direct electronic molecular materials design. In this study, our main focus was on the influence of *n* on the extent of π–π stacking interactions. We found that π–π interactions tend to dominate in high-energy crystals, apart from when *n* = 5–7 where there is little to no correlation, with a minimum when *n* = 6. As a high degree of π–π stacking can give rise to improved charge-transport properties,^74−77^ this result suggests that [5]–[7]helicene and their derivatives may be a fruitful avenue of exploration for developing functional organic electronics. This observation is further supported by the observation of chiral columns predominant in derivatives of either [6] or [7]helicene.^72,73,78,79^ π–π interactions are not the only motifs that result in favorable charge-transport properties, as evidenced by Rice et al. for [6]helicene; as such, future work on fully analyzing the charge-transport properties of structures in each of the CSP landscapes reported here would be beneficial to develop further understanding of structure–property relationships in organic electronics.^26^

## Supporting Information Available

Overlays of experimental and computationally predicted structures and comparison of computational results (PDF)
