# Supplementary material for: Effect of [n]-Helicene Length on Crystal Packing
Source: Cryst Growth Des. 2023 Oct 24;23(12):8909–17. doi: 10.1021/acs.cgd.3c00964 (PMC10704484; doi:10.1021/acs.cgd.3c00964)
Supplement: Supplementary file 1 — cg3c00964_si_001.pdf [file cg3c00964_si_001.pdf]

Supporting Information: The effect of [n]-helicene length on crystal packing

Julia A. Schmidt,[†,¶] Emma H. Wolpert,[†,¶] Grace M. Sparrow,[‡] Erin R. Johnson,[‡] and Kim E. Jelfs[*,†]

†*Department of Chemistry, Imperial College London, Molecular Sciences Research Hub, White City Campus, Wood Lane, London, W12 0BZ, UK*

‡*Department of Chemistry, Dalhousie University, Halifax, Nova Scotia, B3H 4R2, Canada*

¶*Equal contribution*

E-mail: k.jelfs@imperial.ac.uk

Contents

1 **Molecular overlays of experimental determined structures and computationally predicted structures.** **S-3**

2 **Energy rankings of experimentally observed structure** **S-11**

3 **Correlation between lattice energy and the extent of π stacking** **S-12**

1 Molecular overlays of experimental determined structures and computationally predicted structures.

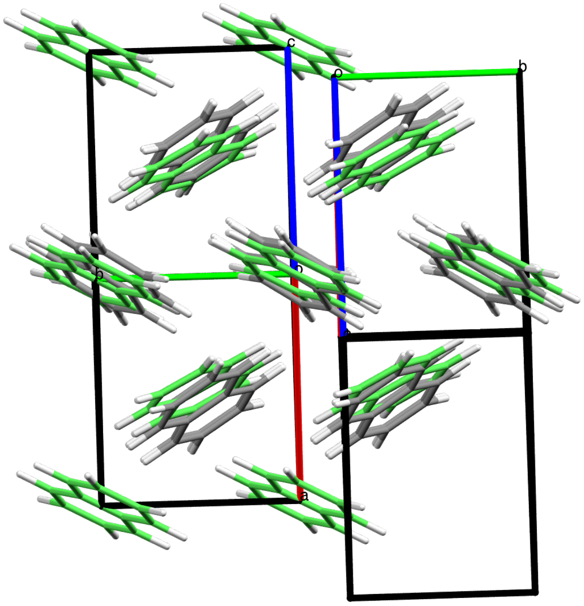

Figure S1: Molecular overlay of the naphthalene X-ray (CSD reference code: NAPTHA18) and the lowest-lying predicted structure. For an overlay of 15 molecules, excluding hydrogens, a RMSD of 0.609 Å,was observed. The experimental (predicted) structure is shown in grey (green).

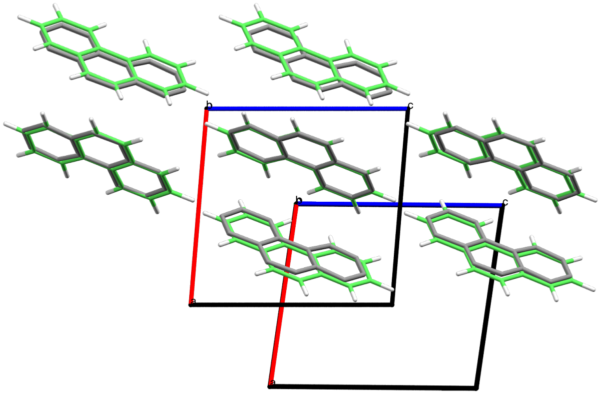

Figure S2: Molecular overlay of the [3]helicene X-ray and the lowest energy predicted structure. For an overlay of 15 molecules, excluding hydrogens, a RMSD = 0.210 Å was observed. The experimentally observed crystal structure parameters have been reported by Trotter in 1963 to be of P21 space group. As the structure is not in the CSD, the crystal structure data provided was used to generate a CIF file, which was then overlaid with predicted structures of similar space group and crystal parameters. The experimental (predicted) structure is shown in grey (green).

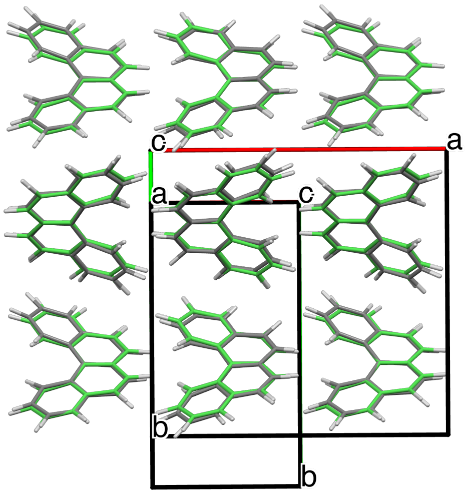

Figure S3: Molecular overlay of the [4]helicene X-ray (CSD reference code: BZPHAN) and the lowest energy predicted structure. For an overlay of 15 molecules, a RMSD = 0.282 Å was observed. The experimental (predicted) structure is shown in grey (green).

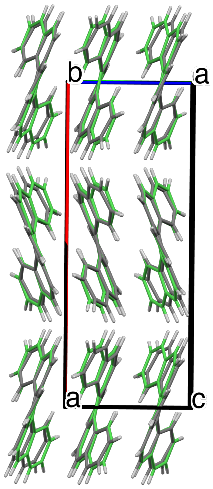

Figure S4: Molecular overlay of the [5]helicene X-ray (CSD reference code: DBPHEN05) and the lowest energy predicted structure. For an overlay of 15 molecules, excluding hydrogens, a RMSD = 0.223 Å was observed. The experimental (predicted) structure is shown in grey (green).

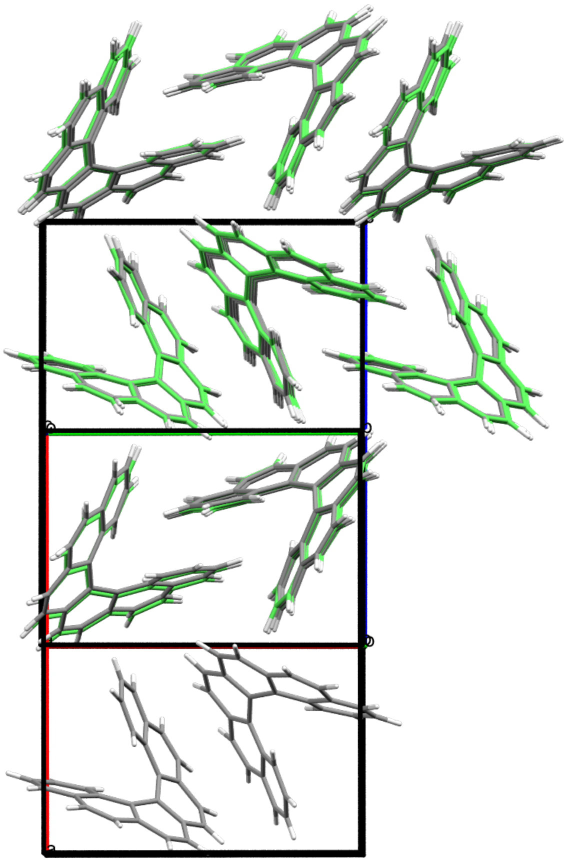

Figure S5: Molecular overlay of the [6]helicene X-ray (CSD reference code: HEXHEL) and the lowest energy predicted structure. For an overlay of 15 molecules, excluding hydrogens, a RMSD = 0.210 Å was observed. The experimental (predicted) structure is shown in grey (green).

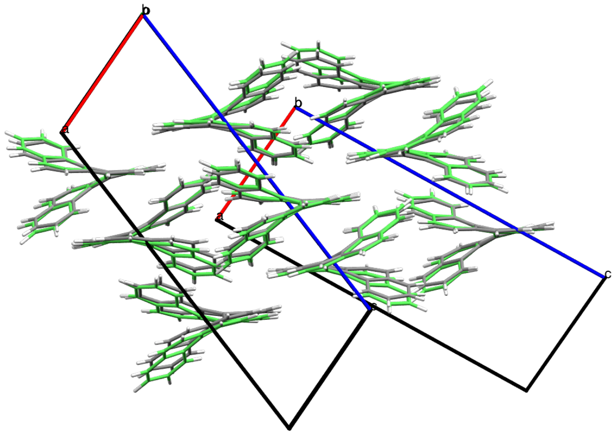

Figure S6: Molecular overlay of the [7]helicene X-ray (CSD reference code: HEXHEL) and the second-lowest energy, but lowest racemic, predicted structure. For an overlay of 15 molecules, excluding hydrogens, a RMSD = 0.315 Å was observed. The experimental (predicted) structure is shown in grey (green).

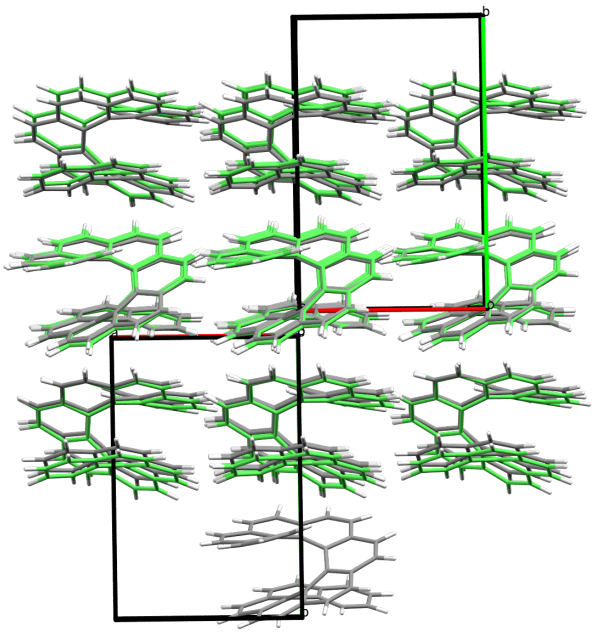

Figure S7: Molecular overlay of the [10]helicene X-ray (CSD reference code: THELIC) and the lowest energy predicted structure. For an overlay of 15 molecules, excluding hydrogens, a RMSD of 0.308 Å was observed. The experimental (predicted) structure is shown in grey (green).

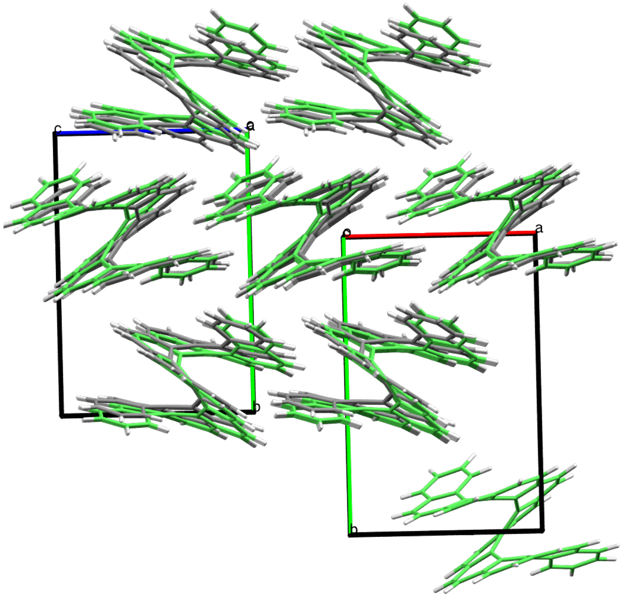

Figure S8: Molecular overlay of the [11]helicene X-ray (CSD reference code: UHELIC) and the lowest energy predicted structure. For an overlay of 15 molecules (at an increased cutoff of 30%), excluding hydrogens, a RMSD of 0.687 Å was observed. The experimental (predicted) structure is shown in grey (green).

2 Energy rankings of experimentally observed structure

Table S1: Relative energy rankings of the $Z' = 1$ experimentally observed structure according to the respective crystal-energy landscape. The relative position across the entire landscape (Total), among all enantiopure and racemic structures is shown.

	Energy ranking DMACRYS			Energy ranking DFT-XDM		
Helicene Length n	Total	Enatiopure	Racemic	Total	Enatiopure	Racemic
Naphthalene (2)	1	-	-	1	-	-
3	2	2	-	1	1	-
4	1	1	-	4	1	-
5	83	-	68	1	-	1
6	2	1	-	1	1	-
7	47	-	44	3	-	1
10	2	2	-	1	1	-
11	6	1	-	1	1	-

3 Correlation between lattice energy and the extent of π stacking

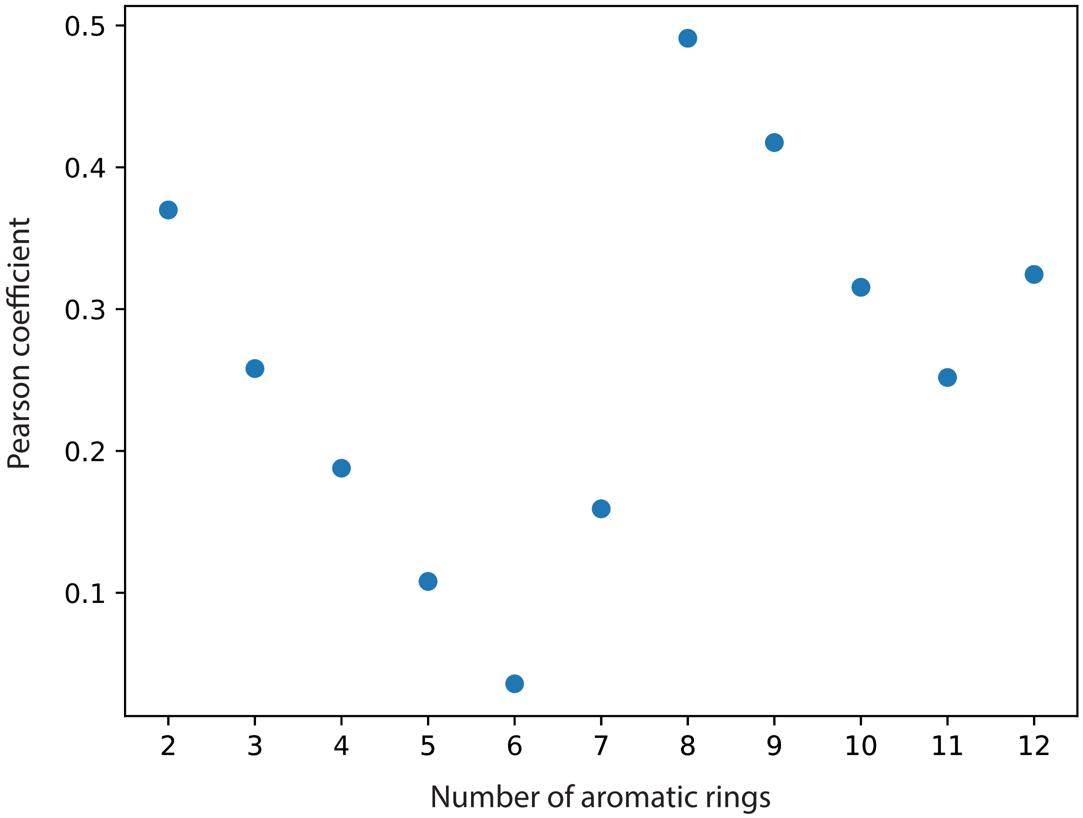

Figure S9: Pearson correlation coefficients between the lattice energy and the extent of π stacking against the number of aromatic rings in the molecule.
